# Drug cross-reactivity between methimazole and propylthiouracil causing recurrent pancreatitis in thyroid storm

**DOI:** 10.1530/EDM-25-0135

**Published:** 2026-04-03

**Authors:** Yuriko Yamazaki, Junichiro Adachi, Yurika Ishihara, Yuto Moriwaki, Wataru Muraoka, Sho Miyashita, Yuki Okuno, Takashi Omura, Yoshihiko Izumida

**Affiliations:** Department of Endocrinology and Diabetes, Saitama Medical Center, Saitama Medical University, Kawagoe, Saitama, Japan

**Keywords:** drug-induced pancreatitis, methimazole, propylthiouracil, drug cross-reactivity, IgG4

## Abstract

**Summary:**

Drug-induced pancreatitis represents 1.1% of acute pancreatitis cases. To date, eight cases of antithyroid drug (ATD)-induced pancreatitis have been reported, including seven associated with methimazole (MMI) and one with carbimazole; however, no cases related to propylthiouracil (PTU) have been reported in the literature. We report a patient with thyroid storm who developed acute pancreatitis during MMI therapy, with recurrence following PTU substitution, suggesting cross-reactivity between antithyroid agents. The patient demonstrated elevated serum immunoglobulin G4 (IgG4) concentrations. The clinical presentation and imaging findings (onset during corticosteroid therapy and absence of specific computed tomography findings) supported a diagnosis of drug-induced rather than IgG4-related autoimmune pancreatitis. However, IgG4-mediated autoimmune hyperactivity may predispose to immunological cross-reactions between antithyroid drugs. In patients with MMI-induced pancreatitis and elevated IgG4 levels, PTU cross-reactivity should be anticipated. Alternative therapies, including potassium iodide, radioactive iodine ablation, or thyroidectomy, warrant consideration when immunological dysfunction is suspected based on elevated IgG4 concentrations.

**Learning points:**

- If abdominal pain, nausea, or vomiting occurs during drug therapy for Graves’ disease, drug-induced pancreatitis should be considered in the differential diagnosis.
- If pancreatitis develops during treatment with MMI, switching to PTU should be approached with caution due to the potential for cross-reactivity – and vice versa.
- In the event that MMI or PTU results in adverse effects, alternative therapeutic modalities should be considered, including radioactive iodine (^131^I) therapy or surgical interventions. These considerations should be informed by the concomitant increase in IgG4 concentration.

## Background

According to a nationwide survey conducted in Japan in 2016, the most common causes of acute pancreatitis were alcohol consumption (32.6%), gallstones (25.8%), and idiopathic (19.1%) and drug-induced (1.1%) etiologies (1). Well-known medications associated with drug-induced pancreatitis include valproic acid and mesalazine. Based on case reports, the European Medicines Agency (EMA) issued a warning in 2019 regarding a potential association between MMI and acute pancreatitis (2). Here, we report the case of a 49-year-old Japanese man who developed acute pancreatitis following treatment with MMI for thyroid storm and experienced recurrence after switching to PTU, suggesting drug-induced pancreatitis related to both agents. Including the present case, only nine cases of antithyroid drug-induced acute pancreatitis have been reported. Among the previous eight cases, four patients were switched from MMI to PTU without recurrence of pancreatitis (3, 4, 5, 6). Unlike those cases, our patient exhibited a different clinical course, indicating the possibility of cross-reactivity between MMI and PTU. Therefore, PTU should be prescribed with caution in patients who have experienced MIP. To our knowledge, no prior reports have described PTU-induced acute pancreatitis, making this the first such case in the literature.

## Case presentation

A 49-year-old Japanese man was referred to our hospital with suspected thyroid storm, presenting with a persistent fever of 39°C for 5 days, along with diarrhea and vomiting that began one day before admission. Before the onset of symptoms, he had no complaints of palpitations, hand tremors, weight loss, general fatigue, or excessive sweating. His medical history included ureteral calculi. He had no history of endocrinopathies or hepatobiliary–pancreatic diseases. He was a non-smoker and had no history of alcohol consumption. His father had been diagnosed with an unspecified parathyroid disorder, but there was no family history of other endocrine, hepatobiliary, or pancreatic diseases. On physical examination, he was alert and oriented, with findings of tachycardia, tachypnea, excessive sweating, and an enlarged thyroid gland.

## Investigation

Blood tests on admission revealed the following – thyrotropin (TSH): 0.01 μIU/mL (reference range (RR): 0.50–5.00), free triiodothyronine (FT3): 14.5 pg/mL (RR: 2.30–4.00), free thyroxine (FT4): >7.77 ng/dL (RR: 0.90–1.70), TSH receptor antibody (TRAb): 8.3 U/L (RR: ≤2.0), thyroglobulin antibody (TgAb): 349 IU/mL (RR: ≤27.0), thyroperoxidase antibody (TPOAb): 108 IU/mL (RR: ≤15.0), and brain natriuretic peptide (BNP): ≤5.8 pg/mL (RR: ≤18.4). Chest X-ray showed no cardiac enlargement or pleural effusion. Electrocardiography revealed tachycardic atrial fibrillation with a heart rate of 165 bpm. Based on these physical findings and laboratory data, a diagnosis of Graves’ disease was made. The patient exhibited no central nervous system symptoms but fulfilled the diagnostic criteria for thyroid storm defined by the Japan Thyroid Association (7), based on the presence of fever (≥38°C), tachycardia (≥130 bpm), and gastrointestinal symptoms (diarrhea, nausea, and vomiting). Furthermore, the Burch–Wartofsky Point Scale (BWPS) score was 55 (≥45), consistent with thyroid storm (8).

## Treatment

**Figure 1:**
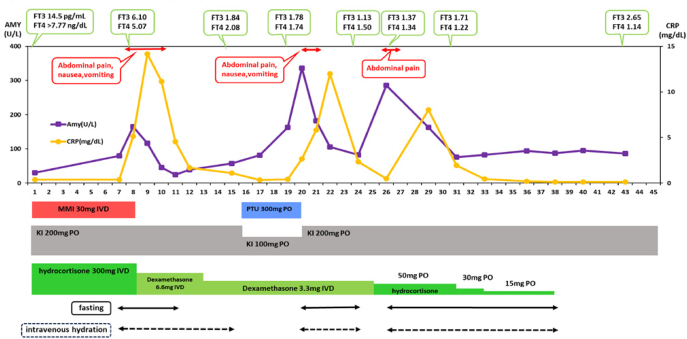
Clinical course during hospitalization. AMY, amylase; CRP, C-reactive protein; FT3, free triiodothyronine; FT4, free thyroxine; MMI, methimazole; PTU, propylthiouracil; KI, potassium iodide; IVD, intravenous drip; PO, per os.

Figure 1 shows the clinical course of the patient during hospitalization. Treatment for thyroid storm was initiated with intravenous methimazole (MMI, 30 mg/day), oral potassium iodide (KI, 200 mg/day), and intravenous hydrocortisone sodium phosphate (300 mg/day). According to the Japanese guidelines, MMI is recommended as the first-line antithyroid drug for Graves’ disease, except during early pregnancy (the organogenesis period, from 4 weeks 0 days to 15 weeks 6 days of gestation) (9). Furthermore, for the management of thyroid storm, intravenous administration of MMI is strongly recommended when gastrointestinal symptoms are present (10). Therefore, intravenous MMI was selected in this case. Tachycardic atrial fibrillation was managed with intravenous landiolol hydrochloride, titrated up to 4 μg/kg/min, and discontinued on hospital day 2 when the heart rate stabilized at 60–80 bpm.

On hospital day 7, the patient developed abdominal pain, nausea, and vomiting. The abdominal pain was dull, non-remitting, and primarily localized to the epigastric region. Laboratory tests revealed a C-reactive protein (CRP) level of 0.39 mg/dL (RR: ≤0.14), white blood cell count of 20,800/μL (RR: 3,300–8,600), and neutrophil percentage of 76.1% (RR: 42.5–75.0%). Serum amylase was within the normal range at 80 U/L (RR: 44–132). Non-contrast abdominal CT revealed no significant findings to explain the abdominal symptoms. Blood cultures showed no bacterial growth. The patient was managed symptomatically with analgesics.

**Figure 2:**
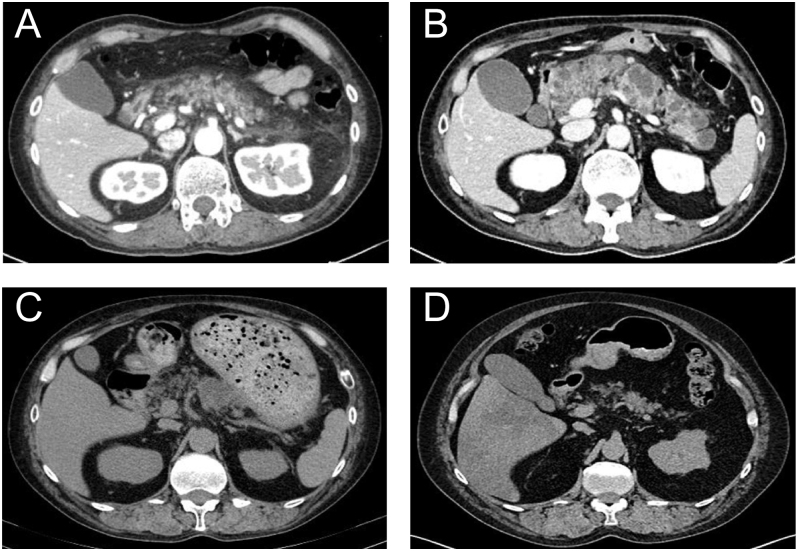
(A) Contrast-enhanced abdominal CT shows pancreatic enlargement and increased attenuation of the peripancreatic fat tissue. (B) Contrast-enhanced abdominal CT shows multiple cystic lesions in the pancreas. (C) Six months later, a non-contrast abdominal CT shows a reduction in the size of the pancreatic cystic lesion. (D) Eighteen months later, non-contrast abdominal CT shows complete resolution of the pancreatic cystic lesion.

On hospital day 8, laboratory tests revealed elevated pancreatic enzymes – amylase: 155 U/L, pancreatic amylase: 136 U/L (RR: 16–52), and lipase: 182 U/L (RR: 17–57). Inflammatory markers were also elevated, with CRP at 5.13 mg/dL, white blood cell count at 28,500/μL, and neutrophils comprising 88.8% of the total count. Contrast-enhanced abdominal CT revealed pancreatic enlargement and peripancreatic inflammatory changes (Fig. 2A). Based on these findings, acute pancreatitis was strongly suspected. The Bedside Index for Severity in Acute Pancreatitis (BISAP) score was 0, indicating mild disease. There was no evidence of hypercalcemia (corrected calcium: 9.5 mg/dL) or hypertriglyceridemia (triglycerides: 49 mg/dL (RR: 50–149)). Abdominal imaging, including contrast-enhanced CT and ultrasonography, showed no signs of gallstones, biliary sludge, biliary dilation, or choledocholithiasis. Serum immunoglobulin G4 (IgG4) was elevated at 210 mg/dL (total IgG: 1,629 mg/dL; RR: 861–1,747). However, in this case, the following three findings were noted that did not strongly support IgG4-related autoimmune pancreatitis: i) the typical capsular margin seen in autoimmune pancreatitis was not clearly visible on CT (11), ii) the patient developed pancreatitis despite being treated with corticosteroids, which typically suppress immune responses, and iii) elevated IgG4 levels are observed in approximately 6.4% of Graves’ disease patients even in the absence of IgG4-related disease (12). Given the temporal relationship – onset occurring 7 days after the initiation of MMI – and the exclusion of other etiologies, MMI-induced pancreatitis was strongly suspected. KI at 200 mg/day was continued. Due to hypokalemia (serum potassium: 2.8 mEq/L), corticosteroid therapy was switched to intravenous dexamethasone sodium phosphate 6.6 mg/day, which lacks mineralocorticoid activity. Following the withdrawal of MMI, there was a rapid improvement in the pancreatic enzymes indicative of pancreatitis, as well as in the inflammatory and physical findings. By hospital day 10, the patient’s abdominal pain had completely resolved.

On hospital day 16, PTU was initiated at 300 mg/day for the treatment of Graves’ disease and KI dose was reduced to 100 mg/day. On hospital day 20, the patient experienced a recurrence of abdominal pain, and laboratory tests showed elevated pancreatic enzymes – amylase 336 U/L (RR: 44–132) and pancreatic amylase: 303 U/L (RR: 16–52), consistent with recurrent pancreatitis. Abdominal CT revealed new multiple pancreatic cysts (Fig. 2B). Following the withdrawal of PTU, there was a swift and substantial improvement in the enzymes associated with pancreatitis, as evidenced by the discontinuation of MMI. Concurrently, inflammatory markers and physical examination findings also demonstrated notable enhancements. By hospital day 22, the abdominal pain had completely resolved. After discontinuation of PTU, the KI dose was increased from 100 to 200 mg/day to achieve adequate control of thyroid function (Fig. 1). Glucocorticoid therapy, which had been initiated at admission for the management of thyroid storm, was gradually tapered and discontinued under close monitoring for potential adrenal insufficiency. Once stable thyroid function was confirmed, the patient was discharged on hospital day 45. Before discharge, definitive surgical treatment with total thyroidectomy was strongly recommended as a curative option; however, because the patient declined surgery, outpatient radioactive iodine (^131^I) therapy (RAI) was planned as an alternative.

Given that certain adverse drug reactions are associated with specific human leukocyte antigen (HLA) alleles, serologic HLA-DNA typing was performed. The following HLA types were identified: HLA-A*02:01, A*02:06; B*40:01, B*15:01; C*03:04, C*-; DRB1*11:01, DRB1*14:54; and DQB1*05:03, DQB1*03:02.

## Outcome and follow-up

After discharge, the patient underwent RAI. The administered dose was 13.5 mCi, the maximum permitted for outpatient treatment in Japan, regardless of thyroid volume (38.95 cm^3^). KI was discontinued for five days before RAI, and the patient adhered to a low-iodine diet for 10 days. Nevertheless, the treatment was unsuccessful, possibly due to the insufficient KI withdrawal period, which may have reduced radioactive iodine uptake (13). Ten months after the initial RAI, the patient developed thyroid eye disease, and a second RAI treatment was therefore abandoned. Although the patient had initially declined surgical intervention, total thyroidectomy was ultimately performed with informed consent, as surgery became the only remaining therapeutic option. Histopathological examination of the thyroid gland revealed no evidence of IgG4-related disease. There was no recurrence of drug-induced pancreatitis, and follow-up non-contrast abdominal CT at 6 months demonstrated a reduction in the size of the pseudo-pancreatic cyst (Fig. 2C). At 18 months, the pseudo-cyst had resolved completely on imaging (Fig. 2D).

## Discussion

The diagnosis of drug-induced acute pancreatitis (DIAP) is generally based on the following criteria (14):

- pancreatitis develops during drug treatment;
- no other identifiable cause of pancreatitis is found;
- discontinuation of the suspected drug leads to symptom resolution; and
- re-administration of the drug results in recurrence of pancreatitis.

In this case, the diagnosis of DIAP was made based on the fulfillment of criteria i) through iii). Criterion iv) was not assessed, given the risk of recurrence of DIAP upon re-administration of MMI, as previously reported (4, 5, 6, 15, 16). The patient exhibited no history of alcohol consumption, and imaging studies excluded the presence of cholelithiasis. Furthermore, the absence of evidence indicative of hypercalcemia or hypertriglyceridemia precludes the secondary pancreatitis.

Drugs may induce pancreatitis by triggering established risk factors, including structural abnormalities (e.g. biliary stasis, liver injury, sphincter of Oddi dysfunction, pancreatic duct obstruction or stenosis, and stones), metabolic disturbances (e.g. hypertriglyceridemia and hypercalcemia), or vascular effects. Certain drugs or their metabolites may exert direct toxic effects on the pancreas (17). Alternatively, an allergic (hypersensitivity) reaction to the drug has been proposed as the pathogenic mechanism in many cases of drug-induced pancreatitis. In such instances, pancreatitis typically develops 1–6 weeks after drug initiation, most often within 30 days. Upon drug rechallenge, recurrence tends to occur rapidly, usually within 1–3 days (18). To date, eight cases of antithyroid drug (ATD)-induced pancreatitis have been reported, including seven associated with methimazole (MMI) and one with carbimazole (Supplementary Table 1 (see section on Supplementary materials given at the end of the article)) (3, 4, 5, 6, 15, 16, 19, 20). The interval from MMI initiation to the onset of pancreatitis ranged from 4 days to 3 months. In six of these cases (five involving MMI and one involving carbimazole), pancreatitis developed within 2 weeks to 1 month after ATD initiation (3, 4, 5, 15, 16, 20). Furthermore, five cases (four associated with MMI and one with carbimazole) occurred upon ATD rechallenge (4, 5, 6, 15, 16), with recurrence reported between 3 h and 5 days after re-administration. These timelines suggest an allergic mechanism in previously reported cases. In the present case, pancreatitis developed approximately one week after the initiation of MMI. After the initial episode resolved, a flare-up occurred five days after starting PTU. This clinical course supports the possibility of an underlying allergic mechanism in this patient as well.

The patient developed drug-induced pancreatitis following treatment with MMI, and the condition recurred after switching to PTU. Of the eight previously reported cases of ATD-induced pancreatitis, four showed no recurrence after switching from MMI to PTU (3, 4, 5, 6). Additionally, a case–control study reported an increased risk of acute pancreatitis associated with continued use of MMI, but not with PTU (21). The findings in this case were in contrast to those reported in the extant literature and represented a rare instance of drug-induced acute pancreatitis caused by a cross-reaction between MMI and PTU. These findings indicated the potential for an underlying immunological disorder.

The patient exhibited an elevated immunoglobulin G4 (IgG4) level of 210 mg/dL, accompanied by a total IgG level of 1,629 mg/dL (RR: 861–1,747). These findings were indicative of multi-organ dysfunction, encompassing pancreatitis and thyroid disease. Consequently, we contemplated the potential involvement of IgG4-related disease. In previously reported cases of MMI-induced pancreatitis, one Japanese patient (3) and one Korean patient (4) were found to carry the HLA DRB1*08:03:02 and DQB1*06:01 allele. In contrast, the patient in this case had HLA-DRB1*11:01 and DRB1*14:54, which do not correspond to the previously reported allele. The observed HLA association did not demonstrate concordance with HLA alleles commonly implicated in IgG4-related diseases and drug-induced pancreatitis (22). Notably, the characteristic histopathological findings of IgG4-RD: i) dense IgG4-positive lymphoplasmacytic infiltrate, ii) focal fibrosis arranged in a storiform pattern, and iii) obliterative phlebitis (23) are not observed in common drug allergies, drug-induced pancreatitis, or thyroid storm. These findings are crucial for distinguishing IgG4-RD from other conditions. However, in resected thyroid specimens, these pathological findings were absent, thus precluding a definitive diagnosis of typical IgG4-RD. Conversely, given the absence of a pancreatic biopsy, the presence of IgG4-related immune abnormalities within the pancreas remains a possibility. Antigen-specific IgG4 antibodies may be pathogenic in certain conditions, which suggests possible involvement of many immunocytes, including neutrophil extracellular traps, M2 macrophages, basophils, plasmablasts, B-cells, and T-cells (Th2-CD4+T, follicular helper T-cells, and CD4+SLAMF7+cytotoxic T-cells), with lineage interaction, playing important roles in the pathogenesis (24).

The proposed diagnostic criteria for IgG4-related thyroid disease include Hashimoto’s thyroiditis, Graves’ disease, and Riedel’s thyroiditis (25). In this case, immunoglobulin G4 (IgG4) levels were elevated at 210 mg/dL (≥135 mg/dL), with total IgG at 1,629 mg/dL (RR: 861–1,747). However, thyroid ultrasonography revealed no hypoechoic lesions, and histopathological examination of the resected thyroid specimen showed no significant lymphocytic or plasma cell infiltration with severe fibrosis (IgG4+ plasma cells >20/HPF and IgG4+/IgG+ plasma cell ratio >30%). Consequently, IgG4-related thyroid disease was excluded.

The diagnosis of IgG4-related disease (IgG4-RD) is typically determined based on a multifaceted evaluation that incorporates both comprehensive diagnostic criteria and organ-specific criteria, as outlined by the Japanese IgG4-RD Study Group (26, 27, 28, 29). A definitive diagnosis of IgG4-RD can be made when any of the established criteria are met. The diagnostic criteria include organ-specific mass or swelling, elevated serum IgG4 levels, and characteristic histopathological findings. Serum IgG4 levels can be elevated in various conditions, including infections, allergies, malignancies, autoimmune diseases, and Castleman disease (26). Therefore, although elevated IgG4 levels are an important clue for diagnosing IgG4-RD, they must be interpreted with caution (30, 31). When technically feasible, a combination of histological confirmation with biochemical and imaging studies is recommended to establish the diagnosis. A classification system with high sensitivity and specificity for IgG4-RD has recently been proposed by Wallace *et al.* (32).

Regarding the association between IgG4-RD and thyroid disease, patients with IgG4-RD often present with markedly positive antithyroid antibodies and a predisposition to hypothyroidism (33). In these patients, an improvement in thyroid function following corticosteroid therapy has been reported. IgG4-rich inflammation has been described in three inflammatory thyroid diseases presenting with systemic or organ-specific manifestations: Hashimoto’s thyroiditis, Graves’ disease, and Riedel’s thyroiditis (34). These thyroid disorders, characterized by IgG4-positive plasma cell-rich inflammation, may share a common underlying pathology with IgG4-RD. Nevertheless, the spectrum of IgG4-related thyroid disorders (IgG4-RTDs) remains a subject of ongoing debate. To clarify the underlying pathophysiology and clinical significance, accumulation of cases based on unified diagnostic criteria for IgG4-RTDs that broadly encompass these thyroid disorders is crucial.

It remains unclear whether increased IgG4 is directly involved in pathogenesis in this case; however, drug cross-reactivity between MMI and PTU might cause recurrent pancreatitis in thyroid storm. In patients with MMI-induced pancreatitis and elevated IgG4, PTU cross-reactivity should be anticipated. Alternative treatments, including potassium iodide, radioactive iodine (^131^I), or thyroidectomy, should be considered when immunological dysfunction is suspected based on elevated IgG4 concentrations.

## Supplementary materials



## Declaration of interest

The authors declare that there is no conflict of interest could be perceived as prejudicing the impartiality of the study reported.

## Funding

This research did not receive any specific grant from any funding agency in the public, commercial, or not-for-profit sector.

## Patient consent

Written informed consent for the publication of their clinical details and clinical images was obtained from the patient.

## Author contribution statement

All authors were involved in the patient’s care. Y Izumida and J Adachi provided guidance and valuable advice for this paper.
