# Supplementary material for: Drug cross-reactivity between methimazole and propylthiouracil causing recurrent pancreatitis in thyroid storm
Source: Endocrinol Diabetes Metab Case Rep. 2026 Apr 3;2026(2):EDM250135. doi: 10.1530/EDM-25-0135 (PMC13052769; doi:10.1530/EDM-25-0135)
Supplement: Supplementary file 1 [file supplementary_materials.pdf]

**Reference**	**Age/sex**	**Ethnicity**	**Diagnosis**	**ATD dose (mg)**	**Interval between the start of ATD and the development of pancreatitis**	**Rechallenge dose (mg)**	**Interval between the start of ATD rechallenge and recurrence of pancreatitis**	**Clinical presentation [a]**	**Laboratory findings [a]**	**Interval between elevation and normalization of pancreatic enzyme levels [b]**	**CT findings of acute pancreatitis [a]**	**Alternative treatment**	**Pancreatitis caused by alternative drugs**	**Interval between the start of alternative drugs and recurrence of pancreatitis**	**IgG4/IgG (mg/dL)**	**HLA**
Taguchi, 1999 [5]	66/F	Japanese	GD	MMI 30	3 weeks	10	3 hours	fever, abdominal pain	WBC 8600/μL, CRP 4.0 mg/dL, amylase 3009 U/L, lipase 1059 U/L	amylase 6 days, lipase 10 days, elastase-1 14 days	N	PTU 300 mg	N	-	NA	A:26/-, B: 62/39, C: w3/w7, DR: 4/14
Marazuela,2002 [15]	33/F	NA	GD	carbimazole 45	1 month	10	24 hours	abdominal pain, weakness, vomiting	WBC 18300/μL, amylase 454 U/L, lipase 2280 U/L	NA	Y	RI	-	-	NA	NA
Yang, 2012 [6]	18/F	Chinese	GD	MMI 20	4 days	10	a few hours	fever, abdominal pain, nausea, vomiting	WBC 6990/μL, amylase 48 U/L, lipase 484 U/L	NA	N	RI→PTU	N	-	NA	NA
Abraham, 2012 [19]	80/F	Caucasian	NA	MMI 10	3 months	-	-	abdominal pain	WBC normal, amylase 371 IU/L, lipase 581 IU/L	lipase 4 days	Y	No alternative	-	-	NA	NA
Jung, 2014 [4]	51/M	Korean	GD	MMI 20	2 weeks	10	5 hours	fever, chill, abdominal pain	WBC 5460/μL, CRP 4.67 mg/dL, amylase 86 IU/L, lipase 86 IU/L	amylase 17 days	Y	PTU 150 mg	N	-	34.9/901.5	DRB1*08:03, DQB1*06:01
Agito, 2015 [16]	51/F	Caucasian	MNG	MMI 10	3 weeks	10	5 days	fever, abdominal pain, diarrhea	lipase 1780 U/mL	lipase 10 days	Y	RI	-	-	NA	NA
Kikuchi, 2019 [20]	76/F	Japanese	GD	MMI 10	19 days	-	-	fever, nausea	WBC 7000/μL, CRP 3.4 mg/dL, amylase 369 IU/L, lipase 1060 U/L	lipase 3 days	Y	KI 200 mg	N	-	38/1367	NA

Case report template © 2021 Bioscientifica Ltd

Yoshimura, 2022[3]	72/F	Japanese	GD	MMI 15	2 weeks	-	-	fever, abdominal pain, nausea, diarrhea	WBC 6400/µL, CRP 9.97 mg/dL, amylase 212 U/L, lipase 923 U/L, elastase-1 1537 ng/dL	amylase 26 days, lipase 98 days, elastase-1 161 days	N	PTU 300 mg	N	-	114/NA	A*02:07:01-A*31:01:02, B*40:01-B*46:01:01, C*01:02-C*03:04:01, DRB1*08:03:02-DRB1*09:01:02
Current case	49/M	Japanese	GD, Thyroid storm	MMI 30	1 week	-	-	abdominal pain, nausea, vomiting	WBC 28500/µL, CRP 5.13 mg/dL, amylase 155 U/L, lipase 182 U/L	amylase 4 days	Y	PTU 300 mg	Y	5 days	210/1629	A*02:01-A*02:06, B*40:01-B*15:01, C*03:04-C*-, DRB1*11:01-DRB1*14:54, DQB1*05:03-DQB1*03:02

SupplementaryTable 1.

Reported cases of drug-induced acute pancreatitis associated with antithyroid drugs (ATDs)

Abbreviations: ATD, antithyroid drug; CRP, C-reactive protein; CT, computed tomography; F, female; GD, Graves' disease; HLA, human leukocyte antigen; IgG, immunoglobulin G; KI, potassium iodide; M, male; MMI, methimazole; MNG, multinodular goiter; N, no; NA, not available; PTU, propylthiouracil; RI, radioiodine; WBC, white blood cell; Y, yes.

a Clinical presentation, laboratory findings, and CT findings of acute pancreatitis at the initial onset of ATD-induced pancreatitis.
The laboratory findings reported by Yang [6] correspond to the second episode of pancreatitis among four episodes, as data from the initial onset were unavailable.

b Interval between the elevation and normalization of pancreatic enzyme levels at the onset of ATD-induced pancreatitis.
In cases involving drug re-challenge, the duration from enzyme elevation to normalization during recurrent episodes of pancreatitis is shown (Taguchi [5], Jung [4], Agito [16]).

Case report template © 2021 Bioscientifica Ltd
